# Aqua­(4-nitro­phthalato-κ*O* ^1^)bis­[2-(1*H*-pyrazol-3-yl-κ*N* ^2^)pyridine-κ*N*]­mangan­ese(II) hemihydrate

**DOI:** 10.1107/S1600536810045952

**Published:** 2010-11-13

**Authors:** Lei Ni, Ji-Li Zhao, Hong Wei

**Affiliations:** a College of Chemistry and Biology, Beihua University, Jilin 132013, People’s Republic of China

## Abstract

In the title compound, [Mn(C_8_H_3_NO_6_)(C_8_H_7_N_3_)_2_(H_2_O)]·0.5H_2_O, the Mn^2+^ ion is octa­hedrally coordinated by two 2-(1*H*-pyrazol-3-yl)pyridine ligands, one 4-nitro­phthalate ligand and one coordinated water mol­ecule leading to an overall MnN_4_O_2_ coordination environment. The two 2-(1*H*-pyrazol-3-yl)pyridine ligands, which deviate from planarity by 0.0187 (2) and 0.0601 (2) Å, make a dihedral angle of 81.90 (6)°. An intra­molecular N—H⋯O hydrogen bond occurs. Inter­molecular π–π stacking inter­actions with a face-to-face separation of 3.61 (1) Å between the 2-(1*H*-pyrazol-3-yl)pyridine ligands is observed. Additionally, O—H⋯O hydrogen bonding involving the uncoordinated water (which is situated on an inversion center), coordinated water mol­ecules and 2-(1*H*-pyrazol-3-yl)pyridine ligands leads to a three-dimensional network in the crystal structure.

## Related literature

For the use of 4-nitro-phthalic acid for metal-organic frameworks, see: Xu *et al.* (2009 ▶); Guo & Guo (2007 ▶).
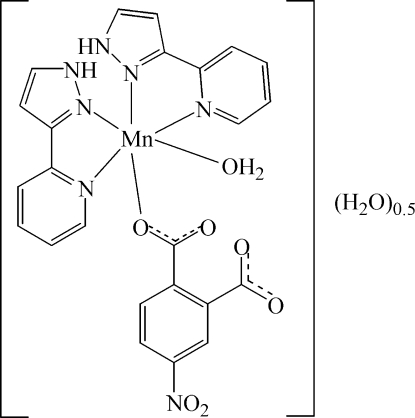


## Experimental

### 

#### Crystal data

[Mn(C_8_H_3_NO_6_)(C_8_H_7_N_3_)_2_(H_2_O)]·0.5H_2_O
*M* *_r_* = 581.41
Triclinic, 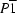

*a* = 10.5996 (7) Å
*b* = 11.2654 (7) Å
*c* = 11.9493 (7) Å
α = 96.275 (2)°
β = 112.485 (2)°
γ = 96.902 (2)°
*V* = 1289.94 (14) Å^3^
*Z* = 2
Mo *K*α radiation
μ = 0.57 mm^−1^
*T* = 294 K
0.12 × 0.10 × 0.08 mm

#### Data collection

Bruker APEXII CCD diffractometer
Absorption correction: multi-scan (*SADABS*; Bruker, 2001 ▶) *T*_min_ = 0.935, *T*_max_ = 0.956
13775 measured reflections
4492 independent reflections
4112 reflections with *I* > 2σ(*I*)
*R*_int_ = 0.016

#### Refinement

*R*[*F* ^2^ > 2σ(*F* ^2^)] = 0.038
*wR*(*F* ^2^) = 0.107
*S* = 1.00
4492 reflections
364 parameters
3 restraints
H atoms treated by a mixture of independent and constrained refinement
Δρ_max_ = 0.67 e Å^−3^
Δρ_min_ = −0.56 e Å^−3^

### 

Data collection: *APEX2* (Bruker, 2004 ▶); cell refinement: *SAINT-Plus* (Bruker, 2001 ▶); data reduction: *SAINT-Plus*; program(s) used to solve structure: *SHELXS97* (Sheldrick, 2008 ▶); program(s) used to refine structure: *SHELXL97* (Sheldrick, 2008 ▶); molecular graphics: *SHELXTL* (Sheldrick, 2008 ▶); software used to prepare material for publication: *SHELXTL*.

## Supplementary Material

Crystal structure: contains datablocks I, global. DOI: 10.1107/S1600536810045952/im2225sup1.cif

Structure factors: contains datablocks I. DOI: 10.1107/S1600536810045952/im2225Isup2.hkl

Additional supplementary materials:  crystallographic information; 3D view; checkCIF report

## Figures and Tables

**Table 1:** Hydrogen-bond geometry (Å, °)

*D*—H⋯*A*	*D*—H	H⋯*A*	*D*⋯*A*	*D*—H⋯*A*
O1*W*—H2*W*⋯O4^i^	0.82 (2)	1.80 (2)	2.615 (2)	171 (3)
N1—H1*A*⋯O1	0.86	1.86	2.644 (3)	152

Symmetry code: (i) 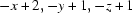.

**Fig. 1.**
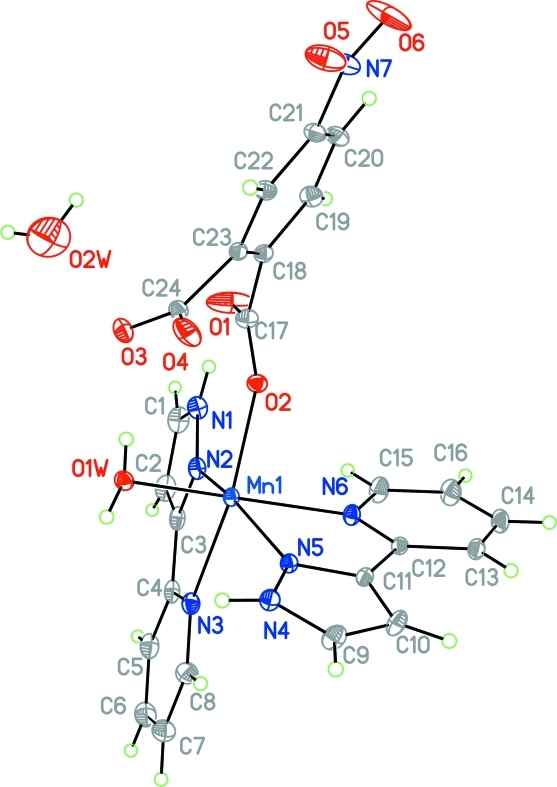
Molecular structure of the title compound with the atom-labelling scheme. Displacement ellipsoids are drawn at the 30% probability level; H atoms are given as spheres of arbitrary radius.

**Fig. 2.**
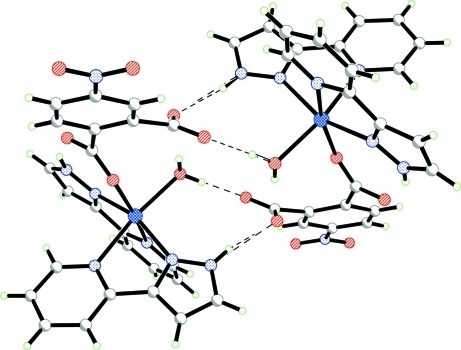
Crystal packing of the title compound, displayed with hydrogen bonds as dashed lines.

## supplementary crystallographic information

### Comment

The synthesis of metal-organic frameworks (MOFs) has attracted continuous research interest not only because of their appealing structural and topological novelty, but also due to their unusual optical, electronic, magnetic, and catalytic properties, as well as their potential medical application (Xu *et al.* (2009); Guo & Guo (2007)). Here, we describe the synthesis and structural characterization of the title compound.

Single crystal X-ray diffraction analysis revealed that the asymmetric unit of the title compound, [(Mn(C_8_H_7_N_3_)_2_(C_8_H_3_NO_6_)(H_2_O)] × 0.5 H_2_O, consists of one Mn^2+^ ion that is octahedrally coordinated by two 2-(1*H*-pyrazol-3-yl)-pyridine ligands, one 4-nitro-phthalato ligand and one coordinated water molecule leading to an overall MnN_4_O_2_ coordination environment (Figure 1). Deviations of the two 2-(1*H*-pyrazol-3-yl)-pyridine moieties from planarity are 0.0187 (2) and 0.0601 (2) Å, respectively. The dihedral angle between the two 3-(2-pyridyl)-1*H*-pyrazole planes is 81.90 (6)°. The Mn-N and Mn-O bond distances are in the range of 2.200 (2)—2.359 (2) and 2.126 (2)—2.162 (2) Å, respectively. Intermolecular π-π stacking interactions with a face-to-face separation of 3.61 (1) Å between the 2-(1*H*-pyrazol-3-yl)-pyridine ligands is observed. Additionally, extensive hydrogen bonding involving solvent water (which are situated at a crystallographic center of inversion), coordinated water molecules and 2-(1*H*-pyrazol-3-yl)-pyridine ligands leads to a three dimensional network in the crystal structure (Figure 2).

### Experimental

A mixture of manganese sulfate hydrate (0.33 mmol, 0.050 g), 2-(1*H*-pyrazol-3-yl)-pyridine (0.32 mmoL, 0.05 g), and 4-nitrophthalic acid (0.24 mmoL, 0.05 g), gadolinium(III) nitrate pentahydrate (0.12 mmoL, 0.05 g), and 14 ml H_2_O was sealed in a 25 ml Teflon-lined stainless steel autoclave at 433 K for three days. Pink crystals suitable for the X-ray experiment were obtained after cooling down to room temperature (yield: 76%). Anal. Calc. for C_48_H_40_Mn_2_N_14_O_15_: C 49.53, H 3.44, N 16.85%; Found: C 49.36, H 3.32, N 16.72%.

### Refinement

All hydrogen atoms bound to carbon were refined using a riding model with C—H = 0.93 Å and *U*_iso_ = 1.2*U*_eq_ (C). The H atoms of the coordinated water molecule were located from difference density maps and were refined with d(O—H) = 0.83 (2) Å, and with a fixed *U*_iso_ of 0.80 Å^2^. Refinement of the H atoms of lattice water did not result in a reasonable model since there is only 0.5 water situated at a crystallographic center of inversion. Hydrogen positions would therefore have to be split. Hence corresponding hydrogen positions were excluded from the final refinement.

### Crystal data

**Table:**

[Mn(C_8_H_3_NO_6_)(C_8_H_7_N_3_)_2_(H_2_O)]·0.5H_2_O	*Z* = 2
*M**_r_* = 581.41	*F*(000) = 594
Triclinic, *P*1	*D*_x_ = 1.497 Mg m^−^^3^
Hall symbol: -P 1	Mo *K*α radiation, λ = 0.71073 Å
*a* = 10.5996 (7) Å	Cell parameters from 4492 reflections
*b* = 11.2654 (7) Å	θ = 1.9–25.0°
*c* = 11.9493 (7) Å	µ = 0.57 mm^−^^1^
α = 96.275 (2)°	*T* = 294 K
β = 112.485 (2)°	Block, pink
γ = 96.902 (2)°	0.12 × 0.10 × 0.08 mm
*V* = 1289.94 (14) Å^3^	

### Data collection

**Table:**

Bruker APEXII CCD diffractometer	4492 independent reflections
Radiation source: fine-focus sealed tube	4112 reflections with *I* > 2σ(*I*)
graphite	*R*_int_ = 0.016
phi and ω scans	θ_max_ = 25.0°, θ_min_ = 1.9°
Absorption correction: multi-scan (*SADABS*; Bruker, 2001)	*h* = −12→12
*T*_min_ = 0.935, *T*_max_ = 0.956	*k* = −13→12
13775 measured reflections	*l* = −14→14

### Refinement

**Table:**

Refinement on *F*^2^	Primary atom site location: structure-invariant direct methods
Least-squares matrix: full	Secondary atom site location: difference Fourier map
*R*[*F*^2^ > 2σ(*F*^2^)] = 0.038	Hydrogen site location: inferred from neighbouring sites
*wR*(*F*^2^) = 0.107	H atoms treated by a mixture of independent and constrained refinement
*S* = 1.00	*w* = 1/[σ^2^(*F*_o_^2^) + (0.065*P*)^2^ + 0.6953*P*] where *P* = (*F*_o_^2^ + 2*F*_c_^2^)/3
4492 reflections	(Δ/σ)_max_ = 0.001
364 parameters	Δρ_max_ = 0.67 e Å^−^^3^
3 restraints	Δρ_min_ = −0.56 e Å^−^^3^

### Special details

**Table:**

Geometry. All e.s.d.'s (except the e.s.d. in the dihedral angle between two l.s. planes) are estimated using the full covariance matrix. The cell e.s.d.'s are taken into account individually in the estimation of e.s.d.'s in distances, angles and torsion angles; correlations between e.s.d.'s in cell parameters are only used when they are defined by crystal symmetry. An approximate (isotropic) treatment of cell e.s.d.'s is used for estimating e.s.d.'s involving l.s. planes.
Refinement. Refinement of *F*^2^ against ALL reflections. The weighted *R*-factor *wR* and goodness of fit *S* are based on *F*^2^, conventional *R*-factors *R* are based on *F*, with *F* set to zero for negative *F*^2^. The threshold expression of *F*^2^ > σ(*F*^2^) is used only for calculating *R*-factors(gt) *etc*. and is not relevant to the choice of reflections for refinement. *R*-factors based on *F*^2^ are statistically about twice as large as those based on *F*, and *R*- factors based on ALL data will be even larger.

### Fractional atomic coordinates and isotropic or equivalent isotropic displacement parameters (Å^2^)

**Table:**

	*x*	*y*	*z*	*U*_iso_*/*U*_eq_	
C1	0.7173 (4)	0.2731 (3)	0.9226 (3)	0.0712 (8)	
H1	0.6343	0.2568	0.9323	0.085*	
C2	0.8448 (4)	0.3131 (3)	1.0143 (3)	0.0697 (8)	
H2	0.8668	0.3294	1.0983	0.084*	
C3	0.9359 (3)	0.3247 (2)	0.9554 (2)	0.0518 (6)	
C4	1.0853 (3)	0.3638 (2)	1.0005 (2)	0.0537 (6)	
C5	1.1703 (4)	0.4045 (3)	1.1239 (2)	0.0695 (8)	
H5	1.1328	0.4073	1.1828	0.083*	
C6	1.3098 (4)	0.4403 (3)	1.1578 (3)	0.0831 (10)	
H6	1.3680	0.4673	1.2401	0.100*	
C7	1.3632 (4)	0.4362 (3)	1.0692 (3)	0.0802 (9)	
H7	1.4576	0.4607	1.0903	0.096*	
C8	1.2730 (3)	0.3948 (3)	0.9483 (3)	0.0670 (7)	
H8	1.3090	0.3916	0.8884	0.080*	
C9	1.3004 (3)	0.2422 (3)	0.5695 (3)	0.0635 (7)	
H9	1.3674	0.2662	0.5403	0.076*	
C10	1.2660 (3)	0.1287 (3)	0.5905 (3)	0.0611 (7)	
H10	1.3037	0.0604	0.5786	0.073*	
C11	1.1618 (2)	0.13717 (19)	0.63382 (19)	0.0406 (4)	
C12	1.0839 (2)	0.04615 (18)	0.67283 (18)	0.0404 (4)	
C13	1.0928 (3)	−0.0766 (2)	0.6586 (2)	0.0526 (6)	
H13	1.1490	−0.1049	0.6217	0.063*	
C14	1.0177 (3)	−0.1549 (2)	0.6995 (3)	0.0663 (7)	
H14	1.0218	−0.2372	0.6906	0.080*	
C15	0.9309 (3)	0.0111 (2)	0.7633 (3)	0.0688 (8)	
H15	0.8747	0.0405	0.7995	0.083*	
C16	0.9367 (4)	−0.1107 (3)	0.7536 (3)	0.0761 (8)	
H16	0.8861	−0.1620	0.7834	0.091*	
C17	0.6605 (2)	0.2144 (2)	0.5118 (2)	0.0513 (6)	
C18	0.5813 (2)	0.1929 (2)	0.3746 (2)	0.0427 (5)	
C19	0.4714 (3)	0.0954 (2)	0.3232 (2)	0.0572 (6)	
H19	0.4438	0.0523	0.3746	0.069*	
C20	0.4030 (3)	0.0616 (2)	0.1979 (3)	0.0619 (7)	
H20	0.3294	−0.0034	0.1639	0.074*	
C21	0.4462 (2)	0.1264 (2)	0.1246 (2)	0.0545 (6)	
C22	0.5544 (2)	0.2235 (2)	0.1721 (2)	0.0479 (5)	
H22	0.5820	0.2648	0.1197	0.058*	
C23	0.6216 (2)	0.25915 (18)	0.29779 (19)	0.0400 (4)	
C24	0.7320 (2)	0.3719 (2)	0.3445 (2)	0.0462 (5)	
Mn1	0.97367 (3)	0.28597 (3)	0.71009 (3)	0.03701 (12)	
N1	0.7320 (2)	0.26121 (19)	0.8159 (2)	0.0564 (5)	
H1A	0.6650	0.2366	0.7450	0.068*	
N2	0.8652 (2)	0.29291 (17)	0.83442 (17)	0.0485 (4)	
N3	1.1369 (2)	0.35929 (18)	0.91298 (18)	0.0528 (5)	
N4	1.22077 (19)	0.31285 (17)	0.59823 (18)	0.0474 (4)	
H4	1.2240	0.3884	0.5919	0.057*	
N5	1.13476 (17)	0.25018 (15)	0.63847 (16)	0.0393 (4)	
N6	1.0021 (2)	0.08928 (16)	0.72337 (18)	0.0467 (4)	
N7	0.3749 (3)	0.0899 (3)	−0.0097 (2)	0.0805 (7)	
O1	0.5935 (2)	0.2119 (4)	0.5746 (2)	0.1504 (16)	
O2	0.78795 (15)	0.22449 (14)	0.54996 (14)	0.0475 (4)	
O3	0.7370 (2)	0.44475 (15)	0.43391 (16)	0.0596 (4)	
O4	0.8061 (2)	0.38723 (19)	0.2870 (2)	0.0791 (6)	
O5	0.4122 (3)	0.1463 (3)	−0.0749 (2)	0.1086 (9)	
O6	0.2814 (4)	0.0044 (3)	−0.0487 (3)	0.1654 (18)	
O1W	0.96188 (16)	0.46463 (14)	0.66095 (15)	0.0479 (4)	
O2W	0.5000	0.5000	0.5000	0.208 (3)	
H1W	0.9011 (17)	0.454 (3)	0.5913 (10)	0.080*	
H2W	1.0347 (14)	0.507 (2)	0.670 (2)	0.080*	

### Atomic displacement parameters (Å^2^)

**Table:**

	*U*^11^	*U*^22^	*U*^33^	*U*^12^	*U*^13^	*U*^23^
C1	0.097 (2)	0.0723 (18)	0.0770 (19)	0.0202 (16)	0.0667 (19)	0.0216 (15)
C2	0.109 (2)	0.0717 (18)	0.0557 (16)	0.0283 (17)	0.0556 (18)	0.0216 (13)
C3	0.0855 (18)	0.0398 (11)	0.0455 (12)	0.0208 (11)	0.0388 (12)	0.0118 (9)
C4	0.0838 (18)	0.0380 (12)	0.0446 (12)	0.0213 (11)	0.0277 (12)	0.0100 (9)
C5	0.106 (2)	0.0573 (16)	0.0450 (14)	0.0253 (15)	0.0264 (15)	0.0096 (12)
C6	0.104 (3)	0.0694 (19)	0.0505 (16)	0.0196 (18)	0.0035 (17)	0.0064 (14)
C7	0.073 (2)	0.076 (2)	0.0705 (19)	0.0113 (16)	0.0083 (16)	0.0066 (15)
C8	0.0655 (17)	0.0692 (17)	0.0592 (16)	0.0113 (14)	0.0192 (13)	0.0037 (13)
C9	0.0590 (15)	0.0756 (18)	0.0797 (18)	0.0222 (13)	0.0481 (14)	0.0230 (14)
C10	0.0679 (16)	0.0625 (16)	0.0775 (17)	0.0340 (13)	0.0465 (14)	0.0217 (13)
C11	0.0430 (11)	0.0413 (11)	0.0400 (11)	0.0145 (9)	0.0174 (9)	0.0070 (8)
C12	0.0442 (11)	0.0390 (11)	0.0347 (10)	0.0117 (9)	0.0112 (9)	0.0057 (8)
C13	0.0630 (14)	0.0435 (12)	0.0492 (13)	0.0197 (11)	0.0175 (11)	0.0072 (10)
C14	0.0842 (19)	0.0372 (13)	0.0728 (17)	0.0156 (12)	0.0235 (15)	0.0146 (12)
C15	0.0821 (19)	0.0506 (14)	0.101 (2)	0.0164 (13)	0.0608 (17)	0.0260 (14)
C16	0.092 (2)	0.0478 (15)	0.105 (2)	0.0101 (14)	0.0540 (19)	0.0311 (15)
C17	0.0418 (12)	0.0668 (15)	0.0513 (13)	0.0064 (10)	0.0257 (10)	0.0111 (11)
C18	0.0337 (10)	0.0464 (12)	0.0515 (12)	0.0080 (9)	0.0206 (9)	0.0084 (9)
C19	0.0470 (13)	0.0583 (14)	0.0676 (16)	−0.0026 (11)	0.0259 (12)	0.0176 (12)
C20	0.0428 (13)	0.0561 (15)	0.0722 (17)	−0.0106 (11)	0.0149 (12)	0.0028 (12)
C21	0.0420 (12)	0.0584 (14)	0.0500 (13)	0.0027 (10)	0.0089 (10)	−0.0010 (11)
C22	0.0416 (11)	0.0532 (13)	0.0472 (12)	0.0032 (10)	0.0170 (10)	0.0096 (10)
C23	0.0338 (10)	0.0382 (11)	0.0475 (11)	0.0056 (8)	0.0162 (9)	0.0067 (9)
C24	0.0433 (11)	0.0391 (11)	0.0504 (12)	0.0008 (9)	0.0143 (10)	0.0085 (10)
Mn1	0.0428 (2)	0.03495 (19)	0.03942 (19)	0.00804 (13)	0.02298 (15)	0.00565 (13)
N1	0.0672 (13)	0.0579 (12)	0.0593 (12)	0.0069 (10)	0.0432 (11)	0.0088 (9)
N2	0.0655 (12)	0.0437 (10)	0.0483 (11)	0.0105 (9)	0.0356 (10)	0.0075 (8)
N3	0.0663 (13)	0.0466 (11)	0.0465 (11)	0.0141 (9)	0.0234 (10)	0.0048 (8)
N4	0.0476 (10)	0.0460 (10)	0.0576 (11)	0.0075 (8)	0.0302 (9)	0.0124 (8)
N5	0.0414 (9)	0.0379 (9)	0.0432 (9)	0.0072 (7)	0.0221 (8)	0.0062 (7)
N6	0.0541 (11)	0.0376 (9)	0.0570 (11)	0.0102 (8)	0.0298 (9)	0.0121 (8)
N7	0.0622 (15)	0.0935 (19)	0.0578 (15)	−0.0044 (14)	0.0031 (12)	−0.0036 (14)
O1	0.0515 (13)	0.342 (5)	0.0589 (13)	0.015 (2)	0.0337 (11)	0.016 (2)
O2	0.0402 (8)	0.0541 (9)	0.0470 (8)	0.0068 (7)	0.0177 (7)	0.0041 (7)
O3	0.0743 (12)	0.0413 (9)	0.0559 (10)	−0.0013 (8)	0.0228 (9)	0.0036 (8)
O4	0.0715 (13)	0.0731 (13)	0.0920 (14)	−0.0272 (10)	0.0501 (12)	−0.0099 (11)
O5	0.0866 (17)	0.167 (3)	0.0508 (12)	−0.0142 (17)	0.0190 (12)	0.0042 (14)
O6	0.160 (3)	0.160 (3)	0.0776 (18)	−0.088 (3)	−0.0201 (19)	−0.0018 (18)
O1W	0.0464 (9)	0.0414 (8)	0.0532 (9)	0.0005 (7)	0.0182 (7)	0.0109 (7)
O2W	0.190 (6)	0.278 (8)	0.248 (8)	0.101 (6)	0.165 (6)	0.066 (6)

### Geometric parameters (Å, °)

**Table:**

C1—N1	1.338 (3)	C15—H15	0.9300
C1—C2	1.357 (5)	C16—H16	0.9300
C1—H1	0.9300	C17—O1	1.216 (3)
C2—C3	1.398 (4)	C17—O2	1.235 (3)
C2—H2	0.9300	C17—C18	1.504 (3)
C3—N2	1.332 (3)	C18—C19	1.393 (3)
C3—C4	1.456 (4)	C18—C23	1.398 (3)
C4—N3	1.352 (3)	C19—C20	1.375 (4)
C4—C5	1.390 (4)	C19—H19	0.9300
C5—C6	1.370 (5)	C20—C21	1.370 (4)
C5—H5	0.9300	C20—H20	0.9300
C6—C7	1.378 (5)	C21—C22	1.378 (3)
C6—H6	0.9300	C21—N7	1.473 (3)
C7—C8	1.382 (4)	C22—C23	1.380 (3)
C7—H7	0.9300	C22—H22	0.9300
C8—N3	1.334 (4)	C23—C24	1.511 (3)
C8—H8	0.9300	C24—O4	1.235 (3)
C9—N4	1.337 (3)	C24—O3	1.253 (3)
C9—C10	1.364 (4)	Mn1—O2	2.1255 (15)
C9—H9	0.9300	Mn1—O1W	2.1619 (15)
C10—C11	1.396 (3)	Mn1—N2	2.1995 (17)
C10—H10	0.9300	Mn1—N5	2.2401 (16)
C11—N5	1.338 (3)	Mn1—N6	2.2861 (18)
C11—C12	1.465 (3)	Mn1—N3	2.359 (2)
C12—N6	1.338 (3)	N1—N2	1.339 (3)
C12—C13	1.393 (3)	N1—H1A	0.8600
C13—C14	1.370 (4)	N4—N5	1.347 (2)
C13—H13	0.9300	N4—H4	0.8600
C14—C16	1.364 (4)	N7—O6	1.203 (4)
C14—H14	0.9300	N7—O5	1.203 (4)
C15—N6	1.334 (3)	O1W—H1W	0.820 (12)
C15—C16	1.375 (4)	O1W—H2W	0.82 (2)
			
N1—C1—C2	108.0 (3)	C18—C19—H19	119.5
N1—C1—H1	126.0	C21—C20—C19	118.2 (2)
C2—C1—H1	126.0	C21—C20—H20	120.9
C1—C2—C3	105.2 (2)	C19—C20—H20	120.9
C1—C2—H2	127.4	C20—C21—C22	122.3 (2)
C3—C2—H2	127.4	C20—C21—N7	118.6 (2)
N2—C3—C2	109.7 (3)	C22—C21—N7	119.0 (2)
N2—C3—C4	117.4 (2)	C21—C22—C23	119.7 (2)
C2—C3—C4	132.9 (2)	C21—C22—H22	120.2
N3—C4—C5	121.6 (3)	C23—C22—H22	120.2
N3—C4—C3	115.1 (2)	C22—C23—C18	119.03 (19)
C5—C4—C3	123.3 (2)	C22—C23—C24	117.40 (19)
C6—C5—C4	119.2 (3)	C18—C23—C24	123.52 (19)
C6—C5—H5	120.4	O4—C24—O3	125.3 (2)
C4—C5—H5	120.4	O4—C24—C23	116.6 (2)
C5—C6—C7	119.5 (3)	O3—C24—C23	118.0 (2)
C5—C6—H6	120.2	O2—Mn1—O1W	86.45 (6)
C7—C6—H6	120.2	O2—Mn1—N2	93.74 (7)
C6—C7—C8	118.3 (3)	O1W—Mn1—N2	99.70 (7)
C6—C7—H7	120.9	O2—Mn1—N5	101.41 (6)
C8—C7—H7	120.9	O1W—Mn1—N5	95.17 (6)
N3—C8—C7	123.3 (3)	N2—Mn1—N5	159.38 (7)
N3—C8—H8	118.3	O2—Mn1—N6	89.68 (7)
C7—C8—H8	118.3	O1W—Mn1—N6	166.14 (7)
N4—C9—C10	108.0 (2)	N2—Mn1—N6	93.83 (7)
N4—C9—H9	126.0	N5—Mn1—N6	72.55 (6)
C10—C9—H9	126.0	O2—Mn1—N3	164.44 (7)
C9—C10—C11	105.0 (2)	O1W—Mn1—N3	94.04 (7)
C9—C10—H10	127.5	N2—Mn1—N3	70.82 (8)
C11—C10—H10	127.5	N5—Mn1—N3	94.05 (7)
N5—C11—C10	110.3 (2)	N6—Mn1—N3	93.24 (7)
N5—C11—C12	118.75 (18)	C1—N1—N2	110.7 (2)
C10—C11—C12	131.0 (2)	C1—N1—H1A	124.6
N6—C12—C13	121.9 (2)	N2—N1—H1A	124.6
N6—C12—C11	115.00 (18)	C3—N2—N1	106.40 (18)
C13—C12—C11	123.1 (2)	C3—N2—Mn1	120.74 (17)
C14—C13—C12	119.1 (2)	N1—N2—Mn1	132.63 (15)
C14—C13—H13	120.5	C8—N3—C4	118.0 (2)
C12—C13—H13	120.5	C8—N3—Mn1	126.27 (17)
C16—C14—C13	119.2 (2)	C4—N3—Mn1	115.70 (17)
C16—C14—H14	120.4	C9—N4—N5	111.07 (19)
C13—C14—H14	120.4	C9—N4—H4	124.5
N6—C15—C16	123.2 (3)	N5—N4—H4	124.5
N6—C15—H15	118.4	C11—N5—N4	105.65 (16)
C16—C15—H15	118.4	C11—N5—Mn1	116.41 (13)
C14—C16—C15	118.9 (3)	N4—N5—Mn1	137.92 (13)
C14—C16—H16	120.6	C15—N6—C12	117.8 (2)
C15—C16—H16	120.6	C15—N6—Mn1	125.06 (16)
O1—C17—O2	125.8 (2)	C12—N6—Mn1	116.60 (14)
O1—C17—C18	117.3 (2)	O6—N7—O5	123.3 (3)
O2—C17—C18	116.67 (19)	O6—N7—C21	117.6 (3)
C19—C18—C23	119.6 (2)	O5—N7—C21	119.1 (3)
C19—C18—C17	117.4 (2)	C17—O2—Mn1	142.44 (15)
C23—C18—C17	122.66 (19)	Mn1—O1W—H1W	106 (2)
C20—C19—C18	121.1 (2)	Mn1—O1W—H2W	117 (2)
C20—C19—H19	119.5	H1W—O1W—H2W	115 (2)

### Hydrogen-bond geometry (Å, °)

**Table:**

*D*—H···*A*	*D*—H	H···*A*	*D*···*A*	*D*—H···*A*
O1W—H2W···O4^i^	0.82 (2)	1.80 (2)	2.615 (2)	171 (3)
N1—H1A···O1	0.86	1.86	2.644 (3)	152

Symmetry codes: (i) −*x*+2, −*y*+1, −*z*+1.
